# Dentistry and HIV/AIDS related stigma

**DOI:** 10.1590/S0034-8910.2015049005877

**Published:** 2015-10-19

**Authors:** Jesus Eduardo Elizondo, Ana Cecilia Treviño, Deborah Violant

**Affiliations:** I Programa de Posgrado en Biotecnología. Grupo de investigación en Biofármacos e Ingeniería Biofarmacéutica. Escuela Nacional de Posgrado en Ciencias e Ingeniería. Instituto Tecnológico de Monterrey. Monterrey, México; II Programa de Posgrado en Odontología. Escuela de Doctorado. Universitat Internacional de Catalunya. Barcelona, España; III Departamento de Ciencias Básicas. Escuela Nacional de Medicina. Instituto Tecnológico de Monterrey. Monterrey, México; IV Programa de Pregrado Médico Cirujano Odontólogo. Escuela de Biotecnología y Ciencias de la Salud. Instituto Tecnológico de Monterrey. Monterrey, México

**Keywords:** HIV Long-Term Survivors, Dental Health Services, Health Knowledge, Attitudes, Practice, Prejudice, Social Discrimination, Health Inequalities, Psychometrics, Mexico

## Abstract

**OBJECTIVE:**

To analyze HIV/AIDS positive individual’s perception and attitudes regarding dental services.

**METHODS:**

One hundred and thirty-four subjects (30.0% of women and 70.0% of men) from Nuevo León, Mexico, took part in the study (2014). They filled out structured, analytical, self-administered, anonymous questionnaires. Besides the sociodemographic variables, the perception regarding public and private dental services and related professionals was evaluated, as well as the perceived stigma associated with HIV/AIDS, through a Likert-type scale. The statistical evaluation included a factorial and a non-hierarchical cluster analysis.

**RESULTS:**

Social inequalities were found regarding the search for public and private dental professionals and services. Most subjects reported omitting their HIV serodiagnosis and agreed that dentists must be trained and qualified to treat patients with HIV/AIDS. The factorial analysis revealed two elements: experiences of stigma and discrimination in dental appointments and feelings of concern regarding the attitudes of professionals or their teams concerning patients’ HIV serodiagnosis. The cluster analysis identified three groups: users who have not experienced stigma or discrimination (85.0%); the ones who have not had those experiences, but feel somewhat concerned (12.7%); and the ones who underwent stigma and discrimination and feel concerned (2.3%).

**CONCLUSIONS:**

We observed a low percentage of stigma and discrimination in dental appointments; however, most HIV/AIDS patients do not reveal their serodiagnosis to dentists out of fear of being rejected. Such fact implies a workplace hazard to dental professionals, but especially to the very own health of HIV/AIDS patients, as dentists will not be able to provide them a proper clinical and pharmaceutical treatment.

## INTRODUCTION

HIV epidemic is about to reach its fourth decade. It is considered a relevant public health care problem worldwide, regardless of antiretroviral therapy advances, which has made of this infection a chronic illness. Life quality and expectancy rates of people living with HIV/AIDS (PLWHA) may be compared to those of the general population.^18,23^ Nevertheless, social perception towards PLWHA remains a negative one.^2,12^ Its transmission routes, its implications regarding the most traditional gender roles, and its association in the social imaginary to socially marginalized groups are the cause of stigma and discrimination at different levels.^2,12^

Stigma is a degrading social evaluation or label that is attached to people that exhibits socially undesirable characteristics.^9^ Stigmatization is the social process by which such evaluations or labels and the consequent negative emotional and behavioral responses are generated and sustained. Therefore, originating and shaping social exclusion.^4^

Stigma is sustained by a complex set of factors difficult to address. An existing duality is observed between the social rejection and approval of attitudes and behaviors of people with certain characteristics. In addition, factors such as beliefs and the environment in which stigma is developed, impose the intensity of the rejection or the acceptance of individuals in a certain context.^20^

HIV/AIDS related stigma and discrimination have multiple consequences that affect HIV epidemic development and reinforce existing social inequalities, especially those related to gender roles, sexuality, and ethnicity. The stigma PLWHA go by is an obstacle in their access to health care services and their engagement into the “HIV continuum of care”.^6,12,19^

Evidence-based scientific research shows PLWHA’s needs in oral health care, due to the high incidence of HIV-related oral problems that diminish their quality of life.^1,25^ This study intends to analyze HIV/AIDS positive individual’s perception and attitudes towards the received oral health care.

## METHODS

One hundred and thirty-four subjects (30.0% of women and 70.0% of men) from Nuevo León, Mexico, took part in the study (2014). They filled out a written or online format questionnaire at the non-governmental and community-based organizations members of Nuevo Leon’s Multisector Response Board to HIV, AIDS, and other sexually-transmitted infections (MEMUREIVH). A non-probabilistic and convenience sample including HIV-positive men and women was adopted.

The data collection process lasted 60 days. Prior to the data collection, collaboration was requested to the different non-governmental and community-based organizations members of the MEMUREIVH, to implement the survey in their office location and to their HIV/AIDS positive affiliates. Four properly qualified surveyors visited all sites and applied the written or on-line surveys on the previously agreed dates and time with the aforementioned organizations. There were no cancellations.

An analytical-type structured questionnaire was used (closed-ended questions and multiple response alternatives) in either written or online formats. Self-administered questionnaires have been shown to be proper methods to collect several data, since they are fully anonymous and, unlike interviews, do not involve a face-to-face confrontation, which could possibly lead to false answers.^3^ The following topics were covered: sociodemographic characteristics, perception regarding public and private dental professionals and services, and that regarding the received HIV-related oral health care. Perception of HIV-AIDS-related stigma in the dental office was analyzed through a Likert-type scale.^15^ Following the procedure recommended by Lynn,^17^ a group of reviewers assessed how well the 11 items evaluated the concepts of stigma and discrimination in terms of their accuracy and relevance for both concepts. Such scale consisted of five alternatives that ranged from “never” to “very often”.

A digital survey database was created and submitted to a double-quality control to assure reliability of information. The subjects accessed the survey website (www.encuestas.no-ip.org) to answer the online questionnaire. No log-in data (e.g., internet protocols) were stored in the electronic access. After survey was completed (average of 25 minutes), and as a benefit to all participants, voluntarily, informed, and consented oral examination and oral prophylaxis were offered free by qualified, certified and sensitized dentists.

An exploratory factor analysis was performed to empirically synthesize the variables concerning the perception of HIV/AIDS-related stigma and discrimination. In this analysis, the inclusion of variables was conditioned by a matrix that validates the presence of marked correlations among all of them and ensures that their application is fitting. Items with no statistical significance were disregarded. The analytical criterion to determine the number of covered factors included the ones whose values were shown to have proper internal consistency index through Cronbach’s alpha.^24^ Principal components analysis was used to extract core factors. Prior to the extraction Kaiser-Meyer-Olkin (KMO) measure of sampling adequacy and Bartlett’s test of sphericity were used to assess the suitability of the respondent data for factor analysis. A Varimax rotation method with Kaiser Normalization were performed to facilitate the interpretation of the factor loadings. A k-means clustering procedure was performed after the factor analysis. Once those clusters had been outlined, each of the factors that were used in their differentiation was proven to be statistically significant by an analysis of variance (ANOVA).

All data were analyzed using SPSS software, version 20.0.

The study protocol was approved by the Ethics Committee at the Tecnologico de Monterrey (BIO-ELB-2012-01). Participants signed informed consent forms. The anonymity and confidentiality of written or electronic data were guaranteed.

## RESULTS

The sample consisted of 134 PLWHA (30.0% of women and 70.0% of men). The mean age of women was 41.7 years (standard deviation [SD] = 10.9; range: 22 to 57 years). Median time of HIV diagnosis was five years.. The average education level was 8.4 (SD = 4.1) and all female subjects reported being heterosexual. The mean age of male participants was 34.9 years (SD = 9; range: 22 to 57), and their median time of HIV diagnosis was two years. In regards to men’s education levels, the average was 14 years (SD = 3.5), and most of them (88.4%) reported being homosexual (Table 1).

**Table 1:** Sociodemographic characteristics of the subjects. Nuevo León, Mexico, 2014.

Variable	Women			Men			p^a^

	Mean	SD	Median	Mean	SD	Median	
Age (years)	41.7	10.9	41^b^	34.9	8	34^b^	< 0.0001
HIV (years of diagnose)	6.10	5.51	5^d^	3.19	4.18	2^d^	< 0.001
Education level (years of schooling)	8.4	4.1	9^b^	14	3.5	15^b^	< 0.0001

Sexual orientation	n	%		n		%	
Homosexual	0	0		83		88.3^b,c^	< 0.0001
Bisexual	0	0		9		9.6	
Heterosexual	40	100^b,c^		2		2.2	
Workload							
Full-time	16	40.0		77		81.9^b,c^	< 0.0001
Part-time	0			10		10.6	
Does not work	24	60.0^b,c^		7		7.4	
Monthly income							
≤ 15,000 MXN (1,146 USD)	40	100^c^		65		69.2^b,c^	< 0.0001
≤ 30,000 MXN (2,292 USD)	0	0		22		23.4	
> 30,000 MXN (2,292 USD)	0	0		7		7.5	

^a^ Through ANOVA.

^b^ (p < 0.001) intergroups.

^c^ (p < 0.001) intragroups.

^d^ (p < 0.001) intergroups.

Forty percent of the women included reported working full time, and the remaining ones were unemployed. However, regardless of their job statuses, their monthly incomes exceeded the minimum wage in northeastern Mexico (≤ 15,000 MXN or 1,146 USD) In the case of men, 81.9% of them reported working full time, whereas 10.6% reported working part-time and the remaining 7.4% were unemployed. Out of the total, 69.2% men earned a monthly income of ≤ 15,000 MXN (1,146 USD).

Out of all women, 44.7% reported having had dental appointments at least once a year. On the other hand, half of the men reported having them once a year and the remaining half reported having them two times or more a year. (Table 2).

**Table 2:** Perception regarding private and public oral health care professionals and services. Nuevo León, Mexico, 2014.

Variable	Women		Men		p^a^

	n	%	n	%	
How many times a year do you have dental appointments?					
None	11	28.9	0	0	
Once a year	17	44.7^c^	47	50.0	< 0.001
Twice or more a year	10	26.3	47	50.0	
Do you have appointments in a public or in a private dental office?					
None	11	28.9	0	0	
Public	15	39.5^b^	24	25.5	
Private	12	31.6	70	74.5^b,c^	< 0.001
Do you have appointments with a general or with a specialty dentist?					
None	2	5.7	0	0	
I do not know	13	37.1^b^	25	26.6	
General	11	31.4	38	40.4^b,c^	< 0.001
Specialty	9	25.7	31	33.0	
Among the following options, which is the most similar to the main reason why you select a certain dentist?					
Knowledge	12	32.4^b,c^	25	26.6	
Experience	5	13.5	28	29.8^b,c^	< 0.001
Formality	3	8.1	6	6.4	
Price	5	13.5	11	11.7	
Good chairside manner	7	18.9	7	7.4	
Office appearance	1	2.7	6	6.4	
Office hygiene	2	5.4	8	8.5	
Dentist’s personal appearance	2	5.4	3	3.2	

^a^ Through ANOVA.

^b^ (p < 0.001) intergroups.

^c^ (p < 0.001) intragroups.

In regards to the modalities of services, 39.5% of women used public dental services and 74.5% of men reported using private ones. Whilst 37.1% of women reported not knowing whether they received a general or specialist oral health care service, 40.4% of the men reported having the general one and 33.0%, the specialist oral health service.

The main reasons the women pointed out for choosing a certain dentist were their professional knowledge (32.4%) and good chairside manner (18.9%). Conversely, most men (29.8%) based their choices on the professionals’ length of experience, followed by their knowledge (26.6%).

Regarding the women, 72.5% of them reported they considered the dental offices they went to as being neither safe, clean, nor complying with infection control rules required by health care authorities. The perception from most men (91.5%) regarding their elected offices was another one entirely. Besides that, 84.6% of the women and 91.5% of the men considered that dentist have to be specifically trained to deal with PLWHA (Table 3).

**Table 3:** Perception regarding HIV care in dental appointments. Nuevo León, Mexico, 2014.

Variable	Women		Men		p^a^

	n	%	n	%	
Is the dental office you have appointments in safe, clean, and complying with infection-control regulations?					
Yes	11	27.5	86	91.5^b,c^	< 0.001
No	29	72.5^b,c^	8	8.5	
Do you trust your dentist to keep the confidentiality of the information in your dental record?					
Yes	21	58.3^c^	64	68.1^c^	< 0.001
No	15	41.7	29	30.9	
Do you believe it is important that your dentist be qualified to treat HIV patients?					
Yes	33	84.6^c^	86	91.5^c^	< 0.001
No	6	15.4	8	8.5	
Have you informed your dentist of your HIV status?					
Yes	19	48.7	29	30.9	
No	20	51.3	65	69.2^c^	< 0.001
Do you think it is important that you tell your dentist you live with HIV?					
Yes	24	61.5^c^	74	78.7^c^	< 0.001
No	15	38.5	20	21.3	
I have a right not to disclose my HIV status, and that is why I do not tell it to my dentist:					
Yes	26	65.0^b^	66	70.3^c^	< 0.001
No	14	35.0	28	29.8	
I am afraid to be denied dental care, and that is why I do not tell my dentist I live with HIV:					
Yes	25	62.5^c^	64	68.1^c^	< 0.001
No	15	37.5	30	31.9	
I am afraid of the inconveniences that may arise in dental care, and that is why I do not tell my dentist I have HIV:					
Yes	25	62.5^b,c^	41	43.6	< 0.001
No	15	37.5	53	56.4^b,c^	
Do you think dentists in general are health care professionals who follow ethical principles, and because of that, they treat you like any other patient, regardless of whether you are HIV positive or not?					
Yes	16	43.2	76	80.9^b,c^	< 0.001
No	20	54.1^b^	18	19.1	
I do not know	1	2.7	0	0	
Do you believe HIV/AIDS can be transmitted in dental offices?					
Yes	13	33.3	42	44.7	
No	26	66.7^b^	51	54.3^c^	< 0.001
I do not know	0	0	0	0	
Do you believe your dentist may transmit HIV?					
Yes	5	12.5	16	17.0	
No	34	85.0^c^	78	83.0^c^	< 0.001
I do not know	1	2.5	0	0	
Do you believe you can transmit HIV to your dentist?					
Yes	10	25.6	31	33.0	
No	29	74.4^c^	50	53.2^c^	< 0.001
I do not know	0	0	13	13.8	
Do you believe you can transmit HIV to other people going to the same dental office or clinic?					
Yes	9	22.5	18	19.1	
No	31	77.5^c^	75	79.7^c^	< 0.001
I do not know	0	0	0	0	
Do you believe you can catch a secondary infection during or after being treated in a dental office or clinic because you have HIV?					
Yes	13	33.3	37	39.4	
No	24	61.5^c^	57	60.7^c^	< 0.001
I do not know	2	5.1	0	0	
Do you believe people living with HIV must only seek care in dental offices or clinics which are exclusive to HIV patients?					
Yes	15	38.5	21	23.3	
No	24	61.5^c^	73	77.7^c^	< 0.001
I do not know	0	0	0	0	
Do you believe oral illnesses affect your general state of health?					
Yes	35	87.5^c^	63	67.0^c^	< 0.001
No	5	12.5	31	33.0	
I do not know	0	0	0	0	
How do you appraise your overall oral health?					
Excellent	8	20.5	6	6.4	
Good	28	71.8^c^	40	42.6^c^	< 0.001
Fair	1	2.6	36	38.3	
Poor	2	5.1	12	12.8	
I do not know	0	0	0	0	

HIV: human immunodeficiency virus

^a^ Through ANOVA.

^b^ (p < 0.001) intergroups.

^c^ (p < 0.001) intragroups.

In 58.3% of the cases involving women and in 68.1% of cases involving men, dentists were said to be reliable concerning the confidentiality of the information in their dental records. However, only 48.7% of the women and 30.9% of the men reported disclosing their HIV serodiagnosis to dental professionals. Despite PLWHA (61.5% of the women and 78.7% of the men) considered it was important that their dentists were aware of their HIV-positive condition, they did not disclose it, since they (63.5% of women and 68.1% of men) were concerned they would be denied oral health care or that inconveniences would arise during dental appointment (in this last case, 62.5% of women and 43.6% of men). In turn, 65.0% of the women and 70.3% of the men believed they were entitled to keep from disclosing their HIV/AIDS serodiagnosis to their dentists (Table 3).

Most women (54.1%) considered that their dentists do not take professional ethics into account, and that is why they thought they would not receive the same care a non-HIV patient would receive. Such perception regarding the dental professionals ran contrary to the women’s by most men (80.9%).

Most PLWHA (66.7% of women and 54.3% of men) do not believe HIV can be transmitted in dental office environments; 85.0% of the women and 83.0% of the men do not believe the dental professionals can transmit the virus to their patients, or vice versa (74.4% of women and 53.2% of men) during appointments. Most PLWHA (77.5% of the women and 79.7% of the men) does not believe they can transmit HIV to other people going to the same office or clinic. The same way, 61.5% of the women and 77.7% of the men do not find it necessary for them to go to specific dental offices or clinics due to their HIV-positive status.

In spite of most PLWHA (71.8% of the women and 42.6% of the men) considering they have good oral and dental health, they do not believed (61.5% of the women and 60.7% of the men) they can come down with a secondary infection during or after dental care, due to the fact they are infected with HIV. Nonetheless, they stated (87.5% of the women and 67.0% of the men) that their general state of health could be affected by the lack of dental care if they will suffer from any HIV-related oral manifestations.

Most PLWHA (69.4% of the women and 84.0% of the men) reported never had experienced any discrimination from dentists, nor that dental professionals had denied them care due to their HIV-positive status (79.5% of the women and 90.4% of the men). Most subjects (76.9% of the women and 83.0% of the men) were never given excuses to denied their oral health care service, nor did most of them (69.2% of the women and 86.2% of the men) had their oral care purposefully delayed due to their PLWHA condition, as compared to the other patients (Table 4).

**Table 4:** Perception of the HIV/AIDS-related stigma and discrimination Nuevo León, Mexico, 2014.

Items	Never		Rarely		Occasionally		Often		Very often	

	n (%)		n (%)		n (%)		n (%)		n (%)	

	W	M	W	M	W	M	W	M	W	M
Have you ever been discriminated by a dentist because you live with HIV?	25 (69.4)	79 (84.0)	4 (11.1)	8 (8.5)	4 (11.1)	5 (5.3)	2 (5.6)	1 (1.1)	1 (2.8)	1 (1.1)
Over the last 12 months, how often were you denied dental care for being HIV positive?	31 (79.5)	85 (90.4)	3 (7.7)	7 (7.4)	5 (12.8)	1 (1.1)	0	1 (1.1)	0	1 (1.1)
When you go to a dental office or clinic, has any one given you a reason for denying you dental care services cos you are HIV positive?	30 (76.9)	78 (83.0)	2 (5.1)	10 (10.6)	4 (10.3)	3 (3.2)	3 (7.7)	2 (2.1)	0	1 (1.1)
When you go to a dental office or clinic, does it take you longer to be treated than other patients?	27 (69.2)	81 (86.2)	3 (7.7)	5 (5.3)	6 (15.4)	5 (5.3)	2 (5.1)	2 (2.1)	1 (2.6)	1 (1.1)
When you go to a dental office or clinic, do you notice whispers, glances, or laughter towards you?	31 (81.6)	78 (83)	0	9 (9.6)	4 (10.5)	5 (5.3)	3 (7.9)	2 (2.1)	0	0
Have you ever been made to fell blamed, undermined, or belittled for having HIV whilst being treated in a dental office or clinic?	31 (81.6)	83 (88.2)	1 (2.6)	4 (4.3)	3 (7.9)	5 (5.3)	1 (2.6)	0	2 (5.3)	2 (2.1)
Have you ever been given negative opinions about your lifestyle or sexual behaviors whilst being treated in a dental office or clinic?	30 (78.9)	82 (87.2)	0	6 (6.4)	5 (13.2)	5 (5.3)	2 (5.3)	1 (1.1)	1 (2.6)	0
Have you ever been treated with disdain, indifference, or criticism whilst being treated in a dental office or clinic?	29 (74.4)	82 (87.2)	3 (7.7)	6 (6.4)	4 (10.3)	6 (6.4)	1 (2.6)	0	2 (5.1)	0
Has anyone ever avoided getting in contact with your skin whilst being treated in a dental office or clinic?	29 (78.4)	84 (89.4)	1 (2.7)	7 (7.4)	3 (8.1)	2 (2.1)	2 (5.4)	1 (1.1)	2 (5.4)	0
Has anybody ever behaved in a way to display fear or insecurity at the time they had to dress wounds, apply sutures, injections, or other dental procedures during treatment in a dental office or clinic?	29 (74.4)	82 (87.2)	0	7 (7.4)	7 (17.9)	2 (2.1)	1 (2.6)	2 (2.1)	2 (5.1)	1 (1.1)
Whilst being treated in a dental office or clinic, has anybody ever requested that the materials used in your treatment be discarded, based on the argument that your HIV-positive status offered a higher risk?	29 (76.3)	83 (88.3)	1 (2.6)	7 (7.4)	2 (5.3)	0	2 (5.3)	1 (1.1)	4 (10.5)	3 (3.2)

HIV: human immunodeficiency virus; W: women; M: men

Most subjects (81.6% of the women and 83.0% of the men) never felt being the target of whispers, glances, or laughter during their dental appointments. The majority of them (81.6% of the women and 88.2% of the men) have never received negative opinions about their lifestyles or sexual behaviors either, nor have they been belittled by their dentists or by their staff (74.4% of the women and 87.2% of the men).

Among the subjects, 78.4% of the women and 89.4% of the men reported that never had noticed that dentists or their staff avoided direct contact with them, neither have most of them (74.4% of the women and 87.2% of the men) noticed fear or insecurity by dental professionals during their appointments. Finally, 76.3% of the women and 88.3% of the men reported never had heard any request to discard materials used in their appointments under the argument that they are highly risky patients due to HIV.

The 11 items have shown evidence of proper internal consistency,^24^ as none of them were discarded. A Cronbach’s alpha of 0.942 was obtained. The first factor consisted of variables related to stigma and discrimination experiences perceived by participants during dental appointments; the second one, for variables related to participants’ concern towards dentists or their staff attitudes regarding their HIV serodiagnosis. After factor analysis, a non-hierarchical clustering analysis was conducted. The first group included subjects identified as “users who have not experienced HIV-related stigma and discrimination in dental appointments” (85.0%). The second group was characterized by a group of subjects referred to as “users who have not experienced stigma and discrimination, but feel slightly concerned about dentists or their staffs’ reaction if they knew about their HIV-positive status” (12.7%). The third group comprised “users who had experienced stigma and discrimination, and feel concerned about dentists or their staffs’ reaction if they knew about their HIV-positive status” (2.3%) (Table 5).

**Table 5:** Factorial analysis of the perception of the HIV and AIDS- related stigma and discrimination in dental appointments. Nuevo León, Mexico, 2014.

Items	Means and correlation matrix										
	Mean	1	2	3	4	5	6	7	8	9	10
a	0.338										
b	0.185	0.583									
c	0.308	0.661	0.589								
d	0.346	0.514	0.305	0.579							
e	0.323	0.574	0.462	0.658	0.663						
f	0.431	0.522	0.523	0.567	0.545	0.758					
g	0.285	0.540	0.484	0.541	0.596	0.822	0.713				
h	0.262	0.456	0.431	0.537	0.525	0.776	0.676	0.790			
i	0.238	0.516	0.432	0.554	0.541	0.803	0.741	0.802	0.880		
j	0.308	0.539	0.506	0.543	0.555	0.804	0.747	0.771	0.823	0.819	
k	0.308	0.529	0.405	0.469	0.491	0.470	0.613	0.472	0.495	0.472	0.636

	Rotated component matrix										
Items	Factor 1. Non personal disparagement experiences from dentists or their staff				Factor 2. Non concern with dentists or their staffs’ attitudes				Commonalities		

a	0.286				0.821				0.756		
b	0.207				0.785				0.659		
c	0.368				0.768				0.725		
d	0.552				0.458				0.514		
e	0.822				0.402				0.838		
f	0.719				0.456				0.726		
g	0.834				0.337				0.809		
h	0.890				0.239				0.849		
i	0.891				0.271				0.868		
j	0.831				0.378				0.833		
k	0.422				0.569				0.502		
Eigenvalues	7.049				1.030						
Percentage of explained variance	64.079				9.361						

	Distances between final cluster centers										
Cluster	1. Users who have not experienced HIV-related stigma and discrimination in dental appointments.				2. Users who have not experienced stigma and discrimination, but feel slightly concerned about dentists or their staffs’ reaction regarding their HIV+ serodiagnosis.				3. Users who have experienced HIV-related stigma and discrimination, and feel concerned about dentists and their staffs’ reaction regarding their HIV+ serodiagnosis.		

	85.0% (n = 114)				12.7% (n = 17)				2.3% (n = 3)		
1					9.434				5.292*		
2	9.434								4.394*		
3	5.292				4.394						

Rotation method: Varimax with Kaiser Normalization. Kaiser-Meyer-Olkin measure of sampling adequacy: 0.904. Bartlett’s sphericity test p < 0.0001; it converges to three iterations. Non-hierarchical clustering analysis (K-means Cluster)

* p < 0.001 through ANOVA.

## DISCUSSION

Most PLWHA went to a dental office once or more times a year in search of oral health care, as they recognize that oral diseases affect their overall state of health. The socioeconomic and education-related determinants revealed social inequalities in HIV care.^16^ The women who took part in this study tended to seek general and public dental health care services, whereas male participants in this study, as they were observed to have a higher income, tended to seek for dental private and specialized services.

Even though, men and women agreed that (i) dentists must be qualified to treat PLWHA; (ii) it is important to inform dental professionals about one’s HIV-positive diagnose; and (iii) that dentists are believed to keep their patient’s dental record information confidential. At the end, both men and women decline to disclose their HIV-positive status to dental professionals. Stigma is a social process or personal experience which influences all aspects in one’s life. Thus, the HIV/AIDS diagnose is omitted by people suffering from it, in order to avoid being excluded socially. That is what Goffman calls “concealment”.^9^

Similar to what was shown globally in other studies^14,21,22^ with different population groups affected by HIV/AIDS pandemic, among the main reasons why PLWHA do not disclose their HIV status to dentists are fear of being shunned, inconveniences that may arise in the dentist-patient relationship, and one’s right not to disclose their diagnose.

One of the limitations of this study – typically found in other HIV-related studies – is the fact that it was conducted with a small sample, as most patients do not disclose their HIV-positive status.^14^ Nonetheless, the obtained results provide an overview of opinions and problems that PLWHA have undergone concerning their dentists.

Although the results from this study have shown a low percentage of perceived stigma and discrimination in dental appointments, most PLWHA reported (through their answers to the questionnaire) not disclosing their HIV status to dental professionals, whether because of previous experiences of stigma and discrimination these people were submitted to in other situations or due to their social collective identity within a given context. Such omission may lead to workplace hazards for dentists and their staff, not only in the case of HIV transmission (and the provision of post-exposure prophylaxis), but also in the case of infection with other blood-borne pathogens, such as hepatitis B and C viruses.^7,8^ Likewise, such fact jeopardizes the very health of PLWHA, as dental professionals will not be able to provide proper clinical care,^13^ and might prescribe a drug that could enhance or antagonize with antiretroviral therapy.^5,10,11^

According to the Global Report 2013 of the Joint United Nations Programme on HIV/AIDS, 61.0% of the countries reported the existence of laws against discrimination that protect PLWHA. In Mexico – based on the provisions in the first article of its federal constitution and on the first article, second paragraph, section II of the federal law for preventing and eliminating discrimination – it is illegal to stigmatize and deny rights that are afforded to all other citizens. Consequently, denying oral health care to PLWHA and other patients with infectious diseases is considered to be discrimination, as it violates the Universal Declaration of Human Rights, such as the right to adequate health. Nonetheless, the lack of accessible legal services leads to the frequent neglect of many HIV-related discrimination cases.

Both realities (individuals who chose not to disclose their HIV status and dental professionals who denies care to people with infectious diseases) do not ensure that dental professionals or their patients avoid being exposed to HIV and other pathogens, as individuals living with HIV or other infectious diseases may not be aware of being infected. For that reason, dentists and all health care professionals are responsible for and ethically bound to seek training concerning the treatment of HIV and other diseases in its category. They also have to keep up-to-date regarding gender studies, diversity, and human rights, to keep high professional standards and to ensure all patients are provided dignified and egalitarian care.
