# Supplementary material for: Dentistry and HIV/AIDS related stigma
Source: Rev Saude Publica. 2015 Oct 19;49:79. doi: 10.1590/S0034-8910.2015049005877 (PMC4617436; doi:10.1590/S0034-8910.2015049005877)
Supplement: Translation

# Translation: La odontología y el estigma asociado al VIH

**Authors:** Jesus Eduardo Elizondo, Ana Cecilia Treviño, Deborah Violant

**Affiliations:** I Programa de Posgrado en Biotecnología. Grupo de investigación en Biofármacos e Ingeniería Biofarmacéutica. Escuela Nacional de Posgrado en Ciencias e Ingeniería. Instituto Tecnológico de Monterrey.Monterrey, México; II Programa de Posgrado en Odontología. Escuela de Doctorado. Universitat Internacional de Catalunya. Barcelona, España; III Departamento de Ciencias Básicas. Escuela Nacional de Medicina. Instituto Tecnológico de Monterrey. Monterrey, México; IV Programa de Pregrado Médico Cirujano Odontólogo. Escuela de Biotecnología y Ciencias de la Salud. Instituto Tecnológico de Monterrey. Monterrey, México

**Keywords:** Sobrevivientes de VIH a Largo Plazo, Servicios de Salud Dental, Conocimientos, Actitudes y Práctica en Salud, Prejuicio, Discriminación Social, Desigualdades en la Salud, Psicometría, México

## Abstract

**OBJETIVO:**

Analizar la percepción y las actitudes de las personas que viven con VIH/sida hacia los servicios odontológicos.

**MÉTODOS:**

Participaron 134 voluntarios (30.0% mujeres y 70.0% hombres) de Nuevo León, México (2014), que contestaron un cuestionario estructurado de tipo analítico, auto-administrado y anónimo. Además de las variables sociodemográficas, fueron analizadas la percepción sobre los servicios y los prestadores de servicios odontológicos públicos y privados; igualmente se exploró mediante escala tipo Likert la percepción del estigma asociado al VIH/sida. El análisis estadístico incluyó análisis factorial y de *clusters *no jerárquico.

**RESULTADOS:**

Se presentaron desigualdades sociales en la búsqueda de atención de prestadores y servicios odontológicos públicos y privados. La mayoría ocultó su serodiagnóstico y concordó en que el odontólogo debe capacitarse en la atención del VIH. El análisis factorial reveló dos factores: experiencias de estigma y discriminación en la consulta odontológica y sentimientos de preocupación por la actitud del odontólogo o su personal hacia el serodiagnóstico del paciente. El análisis de *clusters* identificó tres grupos: usuarios que no han experimentado estigma ni discriminación (85.0%); los que no han experimentado estigma ni discriminación, pero sienten una ligera preocupación (12.7%); y finalmente, los que han experimentado estigma y discriminación, y sienten preocupación (2.3%).

**CONCLUSIONES:**

Se presentó un bajo porcentaje de estigma y discriminación en la consulta odontológica; sin embargo, la mayoría de las personas que viven con VIH/sida no revelan al odontólogo su serodiagnóstico por temor al rechazo. Estos hechos plantean un riesgo laboral para el odontólogo, pero especialmente para la propia salud de las personas que viven con VIH/sida, dado que el odontólogo no podrá proporcionar un tratamiento clínico y farmacológico adecuado.

## INTRODUCCIÓN

La epidemia de VIH entra en su cuarta década. Es considerado un importante problema de salud pública en todo el mundo, independientemente de los avances en la terapia antirretroviral que ha hecho de esta infección una enfermedad crónica. La calidad y la esperanza de vida de las personas que viven con VIH/sida (PVVS) podrían equipararse a la del resto de la población.^18,23^ A pesar de esto, la percepción social hacia quienes viven con VIH continua negativa.^2,12^ Sus vías de transmisión, sus implicaciones respecto a los mandatos de género más tradicionales y su asociación con el imaginario social a grupos socialmente excluidos causan estigma y discriminación en distintos ámbitos.^2,12^

El estigma es una evaluación o etiqueta social degradante que se adjunta a quienes exhiben características socialmente indeseables.^9^ La estigmatización es el proceso social por el cual las evaluaciones o etiquetas degradantes y las consecuentes respuestas emocionales y conductuales negativas se generan y se mantienen, dando origen y forma a la exclusión social.^4^

El estigma se sustenta en un complejo conjunto de factores que no son fáciles de resolver. Hay dualidad entre el rechazo y la aprobación social de las actitudes y los comportamientos de las personas con determinadas características. Además, factores como las creencias y el medio ambiente en donde se desarrolla el estigma precisan la magnitud del rechazo o la aprobación de los individuos en determinado contexto social.^20^

El estigma y la discriminación relacionados con el VIH/sida tienen múltiples consecuencias que afectan al desarrollo de la epidemia y refuerzan las desigualdades sociales existentes, en especial las relacionadas con el género, la sexualidad y la etnia. El estigma que sufren las PVVS es un obstáculo para el acceso a los servicios de salud y al modelo del “Continuo del tratamiento del VIH”.^6,12,19^

La investigación científica basada en evidencia muestra la necesidad de las PVVS en recibir atención buco-dental, debido a la alta incidencia de padecimientos orales asociados al VIH que afectan su calidad de vida.^1,25^ El objetivo del presente estudio fue analizar la percepción y las actitudes de las personas que viven con VIH/sida hacia la asistencia odontológica recibida.

## MÉTODOS

En este estudio transversal, participaron 134 PVVS (30.0% mujeres e 70.0% hombres) de Nuevo León, México (2014), los cuales contestaron una encuesta impresa u *on-line*, en las organizaciones no gubernamentales y de base comunitaria, pertenecientes a la Mesa Multisectorial de Respuesta al VIH, el sida y otras Infecciones de Transmisión Sexual (ITS) en Nuevo León, México (MEMUREVIH). Se realizó un muestreo no probabilístico, incidental y la muestra estudio la conformaron mujeres y hombres con serodiagnóstico positivo para VIH.

La recogida de datos ocurrió durante 60 días. Para contactar con las PVVS que frecuentaron alguna de las organizaciones no gubernamentales y de base comunitaria pertenecientes a la MEMUREVIH, se solicitó autorización a los directivos así como su colaboración para aplicar las encuestas en sus oficinas. Cuatro encuestadores debidamente capacitados visitaron todas las organizaciones en día y hora previamente pactados con los directivos de las organizaciones para la aplicación de las encuestas. No hubo pérdidas o rechazos.

Se utilizó un cuestionario estructurado analítico (de pregunta cerrada y alternativas múltiples) con formato impreso u *on-line*. El cuestionario auto-administrado ha demostrado ser un medio adecuado para recopilar información diversa, dado que es totalmente anónimo y a diferencia de las entrevistas, no implica una confrontación cara a cara, lo que eventualmente podría conducir a respuestas falsas.^3^ Los temas explorados fueron: características sociodemográficas, percepción hacia los prestadores y servicios odontológicos públicos y privados, y hacia recibir atención y tratamiento odontológico integral del VIH. Se exploraron la percepción del estigma y discriminación asociados al VIH/sida en la consulta odontológica mediante una escala tipo Likert.^15^ Siguiendo el procedimiento recomendado por Lynn,^17^ un grupo de revisores valoró qué tan bien los 11 ítems evaluaban el concepto de estigma y discriminación en términos de la claridad del ítem y de su relevancia para ambos conceptos. Esta escala constaba de cinco alternativas en gradiente desde “nunca” hasta “muy a menudo”.

Se creó una base de datos digital de la encuesta, que fue sometida a un doble control de calidad para asegurar la fiabilidad en el ingreso de la información. Los participantes accedían a la página web de la encuesta (www.encuestas.no-ip.org) para responder el cuestionario *on-line*. No se almacenó ningún registro de datos (dirección y protocolo de internet, entre otros) en el caso del acceso electrónico. Después de la encuesta (duración media de 25 min), se les ofreció como beneficio a las PVVS participantes la realización de una exploración y profilaxis oral de forma voluntaria, informada, consentida y gratuita, realizado por odontólogos capacitados, sensibilizados y certificados.

Se realizó análisis factorial exploratorio para sintetizar empíricamente las variables relacionadas con la percepción del estigma y discriminación asociados al VIH/sida. En este análisis, la inclusión de las variables fue condicionada por una matriz que convalida la presencia de una notable correlación entre todas las variables y garantiza la idoneidad de la aplicación de un análisis factorial. Ítems sin significación estadística no se incluyeron en el análisis factorial. El criterio analítico para determinar el número de los factores conservados en el análisis fueron aquellos cuyos valores mostraran índices adecuados de consistencia interna mediante la estimación del coeficiente alfa de Cronbach.^24^ El método de extracción de los factores fue el de los componentes principales. Se calculó la medida de adecuación muestral de Kaiser, Meyer y Olkin (KMO) y se realizó la prueba de esfericidad de Barlett para comprobar la conveniencia de este análisis. Se realizó una rotación por el método Varimax con Kaiser para facilitar la interpretación de los resultados. Después del análisis factorial, se realizó un análisis de *clusters* no jerárquico (K-Means Cluster). Una vez delimitados estos grupos o *clusters,* se comprobó que cada uno de los factores utilizados en la diferenciación de los grupos fuera estadísticamente significativo mediante el análisis de la varianza (ANOVA).

Se analizaron los datos del programa de análisis estadístico SPSS^®^ versión 20.0.

El estudio fue aprobado por el Comité de Ética del Tecnológico de Monterrey (BIO-ELB-2012-01). Los participantes firmaron consentimiento informado. Se garantizó el anonimato y la confidencialidad de los datos escritos o electrónicos.

## RESULTADOS

La muestra estudio la conformaron 134 PVVS (30.0% mujeres y 70.0% hombres). La edad media de las mujeres encuestadas fue de 41.7 años (desviación estándar [DE]: 10.9; rango: 22 a 57 años). La mediana de tiempo del diagnóstico del VIH fue cinco años. La edad media de escolarización fue 8.4 años (DE = 4.1) y todas se definieron como heterosexuales. Los hombres encuestados presentaron edad media de 34.9 años (DE = 8; rango: 22 a 57), mediana de tiempo del diagnóstico del VIH, dos años. La edad media de escolarización fue 14 (DE = 3.5) y la mayoría (88.3%) se definió como homosexual (Tabla 1).

**Tabla 1:** Características sociodemográficas de los participantes. Nuevo León, México, 2014.

Variable	Mujeres			Hombres			p^a^

	Media	DE	Mediana	Media	DE	Mediana	
Edad (años)	41.7	10.9	41^b^	34.9	8	34^b^	< 0.0001
VIH (años del diagnóstico)	6.10	5.51	5^d^	3.19	4.18	2^d^	< 0.001
Escolarización (años cursados)	8.4	4.1	9^b^	14	3.5	15^b^	< 0.0001

Orientación sexual	n	%		n		%	
Homosexual	0	0		83		88.3^b,c^	< 0.0001
Bisexual	0	0		9		9.6	
Heterosexual	40	100^b,c^		2		2.2	
Trabajo							
Tiempo completo	16	40.0		77		81.9^b,c^	< 0.0001
Medio tiempo	0			10		10.6	
No trabaja	24	60.0^b,c^		7		7.4	
Ingresos mensuales							
≤ 15,000 MXN (1,146 USD)	40	100^c^		65		69.2^b,c^	< 0.0001
≤ 30,000 MXN (2,292 USD)	0	0		22		23.4	
> 30,000 MXN (2,292 USD)	0	0		7		7.5	

^a^ Mediante ANOVA.

^b^ (p < 0.001) inter-grupos.

^c^ (p < 0.001) intra-grupos.

^d^ (p < 0.001) inter-grupos.

El 40.0% de las mujeres contaba con empleo a tiempo completo y el 60.0% no trabajaba. Sin embargo, independientemente de su situación laboral, recibían un ingreso económico mensual superior al salario mínimo, en el noreste de México, de ≤ 15,000 MXN (1,146 USD). El 81.9% de los hombres trabajaba a tiempo completo, mientras el 10.6% lo hacía a tiempo parcial y el 7.4% no trabajaba. El 69.2% de los hombres recibía un ingreso económico mensual de ≤ 15,000 MXN (1,146 USD).

El 44.7% de las mujeres acudió a la consulta odontológica al menos una vez al año. Por otro lado, el 50.0% de los hombres declaró asistir al menos una vez al año y el 50.0% restante asistió dos o más veces al año (Tabla 2).

**Tabla 2:** Percepción hacia los prestadores y servicios de salud oral públicos y privados. Nuevo León, México, 2014.

Variable	Mujeres		Hombres		p^a^

	n	%	n	%	
¿Cuántas veces al año acudes a consulta odontológica?					
Ninguna	11	28.9	0	0	
Una vez al año	17	44.7^c^	47	50.0	< 0.001
Dos o más veces al año	10	26.3	47	50.0	
¿Acudes con un odontólogo del sector público o privado?					
Ninguno	11	28.9	0	0	
Público	15	39.5^b^	24	25.5	
Privado	12	31.6	70	74.5^b,c^	< 0.001
¿Acudes con un odontólogo de práctica general o de especialidad?					
Ninguno	2	5.7	0	0	
No sé	13	37.1^b^	25	26.6	
General	11	31.4	38	40.4^b,c^	< 0.001
Especialidad	9	25.7	31	33.0	
¿De las siguientes opciones marca la que más se asemeje a la razón principal por la que elijes a un odontólogo?					
Conocimientos	12	32.4^b,c^	25	26.6	
Experiencia	5	13.5	28	29.8^b,c^	< 0.001
Formalidad	3	8.1	6	6.4	
Precio	5	13.5	11	11.7	
Trato amable	7	18.9	7	7.4	
Aspecto del consultorio	1	2.7	6	6.4	
Higiene del consultorio	2	5.4	8	8.5	
Aspecto personal del odontólogo	2	5.4	3	3.2	

^a^ Mediante ANOVA.

^b^ (p < 0.001) inter-grupos.

^c^ (p < 0.001) intra-grupos.

El 39.5% de las mujeres acudió a los servicios odontológicos públicos y el 74.5% de los hombres a los servicios privados. El 37.1% de las mujeres desconocía si la atención odontológica que recibía era general o de especialidad. El 40.4% de los hombres recibía atención odontológica general y el 33.0%, especialidad.

Las razones principales por la que las mujeres elegían un determinado odontólogo fueron los conocimientos del profesional (32.4%) y el trato amable (18.9%). Por el contrario, la mayoría de los hombres (29.8%) basaba su elección en la experiencia del odontólogo, seguido por los conocimientos de éste (26.6%).

El 72.5% de las mujeres consideraba que el consultorio al que acudían no era seguro ni limpio y no contaba con las normas de control de infección requeridas por las instancias de salud pública. La percepción del consultorio o clínica dental al que acudían la mayoría de los hombres (91.5%) era totalmente opuesta. Además, el 84.6% de las mujeres y el 91.5% de los hombres creían que era necesario que el odontólogo estuviese capacitado para brindar atención a las PVVS (Tabla 3).

**Tabla 3:** Percepción hacia la atención y tratamiento del VIH en la consulta odontológica. Nuevo León, México, 2014.

Variable	Mujeres		Hombres		p^a^

	n	%	n	%	
¿El consultorio o clínica dental al que acudes es seguro, limpio y cuenta con las normas de control de infección?					
Si	11	27.5	86	91.5^b,c^	< 0.001
No	29	72.5^b,c^	8	8.5	
¿Crees en la confidencialidad de los expedientes odontológicos?					
Si	21	58.3^c^	64	68.1^c^	< 0.001
No	15	41.7	29	30.9	
¿Crees que sea importante que tu odontólogo esté capacitado para brindar atención a personas que viven con VIH?					
Si	33	84.6^c^	86	91.5^c^	< 0.001
No	6	15.4	8	8.5	
¿Le informaste al odontólogo que vives con VIH?					
Si	19	48.7	29	30.9	
No	20	51.3	65	69.2^c^	< 0.001
¿Consideras que sea importante decirle a tu odontólogo que vives con VIH?					
Si	24	61.5^c^	74	78.7^c^	< 0.001
No	15	38.5	20	21.3	
Estoy en mi derecho de no revelar mi condición de persona que vive con VIH razón por la que no le informo al odontólogo:					
Si	26	65.0^b^	66	70.3^c^	< 0.001
No	14	35.0	28	29.8	
Me preocupa se me niegue la atención odontológica razón por la que no le informo al odontólogo que vivo con VIH:					
Si	25	62.5^c^	64	68.1^c^	< 0.001
No	15	37.5	30	31.9	
Me preocupan los inconvenientes que puedan surgir en el servicio odontológico razón por la que no le informo al odontólogo que vivo con VIH:					
Si	25	62.5^b,c^	41	43.6	< 0.001
No	15	37.5	53	56.4^b,c^	
¿En general los odontólogos(as) son profesionales de la salud que se rigen por la ética profesional y por consiguiente te atienden como a cualquier otro usuario independientemente de que vivas con VIH?					
Si	16	43.2	76	80.9^b,c^	< 0.001
No	20	54.1^b^	18	19.1	
No sé	1	2.7	0	0	
¿Crees que el VIH/sida se pueda trasmitir en el consultorio odontológico?					
Si	13	33.3	42	44.7	
No	26	66.7^b^	51	54.3^c^	< 0.001
No sé	0	0	0	0	
¿Crees que tu odontólogo te puede transmitir el VIH?					
Si	5	12.5	16	17.0	
No	34	85.0^c^	78	83.0^c^	< 0.001
No sé	1	2.5	0	0	
¿Crees que puedas tú trasmitirle el VIH al odontólogo?					
Si	10	25.6	31	33.0	
No	29	74.4^c^	50	53.2^c^	< 0.001
No sé	0	0	13	13.8	
¿Crees que puedas tú trasmitirles el VIH a otras personas que acudan al mismo consultorio o clínica odontológica en el que recibes atención?					
Si	9	22.5	18	19.1	
No	31	77.5^c^	75	79.7^c^	< 0.001
No sé	0	0	0	0	
¿Crees que tú puedas adquirir una infección secundaria, durante o después de la atención en el consultorio o clínica odontológica por vivir con VIH?					
Si	13	33.3	37	39.4	
No	24	61.5^c^	57	60.7^c^	< 0.001
No sé	2	5.1	0	0	
¿Crees que las personas que viven con VIH deban atenderse en consultorios o clínicas odontológicas en donde SOLO se atiendan a personas con VIH?					
Si	15	38.5	21	23.3	
No	24	61.5^c^	73	77.7^c^	< 0.001
No sé	0	0	0	0	
¿Crees que las enfermedades buco-dentales afectan tu estado de salud general?					
Si	35	87.5^c^	63	67.0^c^	< 0.001
No	5	12.5	31	33.0	
No sé	0	0	0	0	
¿Cómo consideras tu estado de salud buco-dental?					
Excelente	8	20.5	6	6.4	
Bueno	28	71.8^c^	40	42.6^c^	< 0.001
Regular	1	2.6	36	38.3	
Malo	2	5.1	12	12.8	
No sé	0	0	0	0	

VIH: virus de inmunodeficiencia humana

^a^ Mediante ANOVA.

^b^ (p < 0.001) inter-grupos.

^c^ (p < 0.001) intra-grupos.

El 58.3% de las mujeres y el 68.1% de los hombres creían en la confidencialidad del expediente odontológico, sin embargo, solo el 48.7% de las mujeres y el 30.9% de los hombres le informaban al odontólogo su serodiagnóstico al VIH. A pesar de que las PVVS (61.5% de las mujeres y 78.7% de los hombres) consideraban importante que el profesional estuviese enterado de que viven con VIH, no lo referían, dado que (62.5% de las mujeres y 68.1% de los hombres) les preocupaba que se les negase la atención o que surgieran inconvenientes durante la consulta odontológica (62.5% de las mujeres y 43.6% de los hombres). A su vez, el 65.0% de las mujeres y el 70.3% de los hombres creían estar en su derecho de no revelar su condición de PVVS al odontólogo (Tabla 3).

La mayoría de las mujeres (54.1%) consideraba que los odontólogos no se rigen por la ética profesional y, por consiguiente, no serían atendidas como cualquier otra usuaria que no viva con VIH. La percepción hacia los odontólogos, por la mayoría de los hombres (80.9%) fue contraria a la de las mujeres.

La mayor parte de las PVVS (66.7% de las mujeres y 54.3% de los hombres) no creían que el VIH se pudiese transmitir en el consultorio odontológico. El 85.0% de las mujeres y el 83.0% de los hombres no creía que los odontólogos pudiesen trasmitirles a sus usuarios el VIH, o viceversa (74.4% de las mujeres y 53.2% de los hombres), durante la atención odontológica. La mayoría de las PVVS (77.5% de las mujeres y 79.7% de los hombres) no creían que pudiesen transmitirles el VIH a terceras personas que acudieran al mismo consultorio o clínica odontológica. De la misma forma, el 61.5% de las mujeres y el 77.7% de los hombres no creían que por vivir con VIH debieran atenderse en consultorios o clínicas odontológicas donde únicamente se atiendan a las PVVS.

A pesar que la mayoría de las PVVS (71.8% de las mujeres y 42.6% de los hombres) considera tener un buen estado de salud buco-dental, no creían (61.5% de las mujeres y 60.7% de los hombres) que por vivir con VIH pudiesen adquirir una infección secundaria durante o después de la atención en el consultorio o clínica odontológica. Sin embargo, dijeron (87.5% de las mujeres y 67.0% de los hombres) que su estado de salud general resultaría afectado de no recibir atención o tratamiento odontológico al presentarse alguna comorbilidad buco-dental asociada al VIH.

La mayoría de las PVVS (69.4% de las mujeres y 84.0% de los hombres) señalaron que nunca han experimentado alguna situación de discriminación por parte de algún odontólogo. Tampoco el odontólogo les ha negado la atención odontológica debido a que viven con VIH (79.5% de las mujeres y 90.4% de los hombres). A la mayoría de los participantes (76.9% de las mujeres y 83.0% de los hombres) nunca le han dado excusas para negarles la atención, ni tampoco a la mayor parte (69,2% de las mujeres y 86.2% de los hombres) nunca le han demorado el servicio odontológico por vivir con VIH, con relación al resto de los usuarios (Tabla 4).

**Tabla 4:** Percepción del estigma y discriminación asociados al VIH y el sida. Nuevo León, México, 2014.

Ítems	Nunca		Casi nunca		De vez en cuando		A menudo		Muy a menudo	
	n (%)		n (%)		n (%)		n (%)		n (%)	
	M	H	M	H	M	H	M	H	M	H
¿Has experimentado alguna situación de discriminación por parte de algún odontólogo por vivir con VIH?	25 (69.4)	79 (84.0)	4 (11.1)	8 (8.5)	4 (11.1)	5 (5.3)	2 (5.6)	1 (1.1)	1 (2.8)	1 (1.1)
¿En los últimos 12 meses ¿Con qué frecuencia se te ha negado la atención odontológica debido a que vives con VIH?	31 (79.5)	85 (90.4)	3 (7.7)	7 (7.4)	5 (12.8)	1 (1.1)	0	1 (1.1)	0	1 (1.1)
¿Cuándo has asistido a un consultorio o clínica odontológica dan o te dieron excusas para negarte el servicio debido a que vives con VIH?	30 (76.9)	78 (83.0)	2 (5.1)	10 (10.6)	4 (10.3)	3 (3.2)	3 (7.7)	2 (2.1)	0	1 (1.1)
¿Cuándo has asistido a un consultorio o clínica odontológica se demoran o han demorado más en atenderte que al resto de los usuarios?	27 (69.2)	81 (86.2)	3 (7.7)	5 (5.3)	6 (15.4)	5 (5.3)	2 (5.1)	2 (2.1)	1 (2.6)	1 (1.1)
¿Cuándo has asistido a un consultorio o clínica odontológica realizan o han realizado murmullos, miradas o risas sobre tu persona?	31 (81.6)	78 (83)	0	9 (9.6)	4 (10.5)	5 (5.3)	3 (7.9)	2 (2.1)	0	0
¿Cuándo has asistido a un consultorio o clínica odontológica se te culpabilizó, deslegitimó, o calificó por vivir con VIH?	31 (81.6)	83 (88.2)	1 (2.6)	4 (4.3)	3 (7.9)	5 (5.3)	1 (2.6)	0	2 (5.3)	2 (2.1)
¿Cuándo has asistido a un consultorio o clínica odontológica opinaron negativamente sobre tu vida y comportamientos sexuales?	30 (78.9)	82 (87.2)	0	6 (6.4)	5 (13.2)	5 (5.3)	2 (5.3)	1 (1.1)	1 (2.6)	0
¿Cuándo has asistido a un consultorio o clínica odontológica te atendieron con disgusto, indiferencia o de manera despectiva?	29 (74.4)	82 (87.2)	3 (7.7)	6 (6.4)	4 (10.3)	6 (6.4)	1 (2.6)	0	2 (5.1)	0
¿Cuándo has asistido a un consultorio o clínica odontológica evitaron el contacto con tu sudor o con tu piel?	29 (78.4)	84 (89.4)	1 (2.7)	7 (7.4)	3 (8.1)	2 (2.1)	2 (5.4)	1 (1.1)	2 (5.4)	0
¿Cuándo has asistido a un consultorio o clínica odontológica mostraron temor o inseguridad al momento de realizarte curaciones, suturas, aplicarte inyecciones y/u otros procedimientos odontológicos?	29 (74.4)	82 (87.2)	0	7 (7.4)	7 (17.9)	2 (2.1)	1 (2.6)	2 (2.1)	2 (5.1)	1 (1.1)
¿Cuándo has asistido a un consultorio o clínica odontológica solicitaron que se desechen los materiales que utilizaron contigo, argumentando el alto riesgo que se tiene debido a que vives con VIH?	29 (76.3)	83 (88.3)	1 (2.6)	7 (7.4)	2 (5.3)	0	2 (5.3)	1 (1.1)	4 (10.5)	3 (3.2)

VIH: virus de inmunodeficiencia humana; M: mujeres; H: hombres

La mayoría (81.6% de las mujeres y 83.0% de los hombres) nunca ha experimentado murmullos, miradas o risas hacia su persona, igualmente a la mayoría (81.6% de las mujeres y 88.2% de los hombres), en la consulta odontológica, nunca se les ha culpabilizado o descalificado por vivir con VIH. La mayor parte (78.9% de las mujeres y 87.2% de los hombres) nunca ha recibido opiniones negativas sobre su vida y comportamientos sexuales ni han recibido un trato despectivo por parte del odontólogo o su personal (74.4% de las mujeres y 87.2% de los hombres).

El 78.4% de las mujeres y el 89.4% de los hombres nunca han percibido que el odontólogo o su personal evita el contacto directo, o la mayoría (74.4% de las mujeres y 87.2% de los hombres), nunca ha percibido temor o inseguridad al momento de brindárseles la atención odontológica. El 76.3% de las mujeres y el 88.3% de los hombres nunca han escuchado que se hubiese solicitado desechar el material empleado durante su atención bajo el argumento de alto riesgo debido al VIH.

Los 11 ítems mostraron índices adecuados de consistencia interna;^24^ por lo que ninguno fue eliminado, obteniéndose el valor del coeficiente alfa de Cronbach de 0.942. El primer factor estuvo compuesto por aquellas variables relacionadas con las experiencias de estigma y discriminación percibidas por los usuarios en la consulta odontológica; y el segundo, por aquellas relacionadas con la preocupación de los usuarios por la actitud del odontólogo o su personal hacia su serodiagnóstico. Tras el análisis factorial, se realizó un análisis de *clusters* no jerárquico. En el primer grupo, se identificó a los individuos como “usuarios que no han experimentado estigma ni discriminación asociados al VIH en la consulta odontológica” (85.0%). El segundo grupo se caracterizó por el conglomerado de individuos denominados “usuarios que no han experimentado estigma ni discriminación, pero sienten una ligera preocupación hacia la reacción del odontólogo o su personal al enterarse de su serodiagnóstico de VIH” (12.7%). El tercer grupo se caracterizó como “usuarios que han experimentado algún tipo de estigma y discriminación, y sienten preocupación por la reacción del odontólogo o su personal al enterarse de su serodiagnóstico de VIH” (2.3%) (Tabla 5).

**Tabla 5:** Análisis factorial de la percepción del estigma y discriminación asociados al VIH y el sida en la consulta odontológica. Nuevo León, México, 2014.

Ítems	Medias y matriz de correlaciones										
	Medias	1	2	3	4	5	6	7	8	9	10
a	0.338										
b	0.185	0.583									
c	0.308	0.661	0.589								
d	0.346	0.514	0.305	0.579							
e	0.323	0.574	0.462	0.658	0.663						
f	0.431	0.522	0.523	0.567	0.545	0.758					
g	0.285	0.540	0.484	0.541	0.596	0.822	0.713				
h	0.262	0.456	0.431	0.537	0.525	0.776	0.676	0.790			
i	0.238	0.516	0.432	0.554	0.541	0.803	0.741	0.802	0.880		
j	0.308	0.539	0.506	0.543	0.555	0.804	0.747	0.771	0.823	0.819	
k	0.308	0.529	0.405	0.469	0.491	0.470	0.613	0.472	0.495	0.472	0.636

	Matriz de componentes rotados										
Ítems	Factor 1. Sin experiencias personales de rechazo por parte del odontólogo o su personal				Factor 2. Sin preocupación por la actitud del odontólogo o su personal				Comunalidades		

a	0.286				0.821				0.756		
b	0.207				0.785				0.659		
c	0.368				0.768				0.725		
d	0.552				0.458				0.514		
e	0.822				0.402				0.838		
f	0.719				0.456				0.726		
g	0.834				0.337				0.809		
h	0.890				0.239				0.849		
i	0.891				0.271				0.868		
j	0.831				0.378				0.833		
k	0.422				0.569				0.502		
Autovalores	7.049				1.030						
Porcentaje de la varianza explicada	64.079				9.361						

	Distancias entre los centros de los conglomerados finales										
Conglomerado	1. Usuarios que no han experimentado estigma ni discriminación asociados al VIH en la consulta odontológica				2. Usuarios que no han experimentado estigma ni discriminación pero sienten una ligera preocupación por la reacción del odontólogo o su personal al enterarse de su serodiagnóstico al VIH				3. Usuarios que han experimentado estigma, discriminación y sienten preocupación por la reacción del odontólogo o su personal al enterarse de su serodiagnóstico al VIH		

	85.0% (n = 114)				12.7% (n = 17)				2.3% (n = 3)		
1					9.434				5.292*		
2	9.434								4.394*		
3	5.292				4.394						

Método de rotación: normalización Varimax con Kaiser. Medida de adecuación muestral Kaiser-Meyer-Olkin: 0,904. Prueba de esfericidad de Bartlett p < 0.0001; converge en tres iteraciones. Análisis de *cluster* no jerárquico (K-means Cluster)

* p < 0.001 mediante ANOVA.

## DISCUSIÓN

La mayor parte de las PVVS visitan una o más veces al año al odontólogo en busca de atención odontológica, pues tienen claro que las enfermedades buco-dentales afectan el estado de su salud general. Los determinantes socioeconómicos y educativos denotaron desigualdades sociales en la atención y tratamiento del VIH.^16^ Las mujeres que participaron en el presente estudio se inclinaron hacia los servicios dentales públicos y la atención odontológica de práctica general, mientras que los hombres que viven con VIH, y con mayor ingreso mensual que las mujeres, en este estudio, se inclinaron por los servicios dentales privados y de especialización.

Pese a que, las mujeres y los hombres concordaron (i) que el odontólogo debe capacitarse en la atención de las PVVS, (ii) que es importante notificar al odontólogo de su serodiagnóstico al VIH y (iii) que existe la confianza en la confidencialidad del expediente odontológico; finalmente, ambos desistieron en notificar al odontólogo su serodiagnóstico al VIH. El estigma es un proceso social o una experiencia personal conexa que influye sobre todos los aspectos sociales del afectado. De esta forma, el diagnostico de VIH/sida es ocultado por la persona que lo padece para tratar de evitar el rechazo social que este le pueda generar; es lo que Goffman llama “encubrimiento”.^9^

Al igual que en otras investigaciones^14,21,22^ alrededor del globo y con distintos grupos poblaciones afectados por la pandemia del VIH/sida, entre las razones principales por las cuales las PVVS no le informan al odontólogo la infección por el VIH, perpetúan, el temor al rechazo, inconvenientes que puedan surgir en la relación odontólogo-paciente y el derecho a no revelar el serodiagnóstico.

Una de las limitaciones de este estudio, común con otros relacionados con el VIH, es el empleo de una muestra relativamente pequeña, debido a que la mayoría no revela su estado serológico.^14^ Sin embargo, los resultados obtenidos ofrecen un panorama de las opiniones y los problemas que las PVVS han experimentado con los odontólogos.

Aun cuando los resultados del presente estudio revelaron bajo porcentaje de estigma y discriminación percibidos en la consulta odontológica, no debemos olvidar que la mayoría de las PVVS encuestadas no comunican al odontólogo que viven con VIH, ya sea por las experiencias de estigma y discriminación vividas por las PVVS participantes o por la identidad social colectiva de éstas en su contexto. Lo anterior plantea un riesgo laboral para el odontólogo y el resto del personal que colabora en la clínica o consultorio odontológico, no sólo para el VIH (y la provisión de la profilaxis post-exposición), sino igualmente para otros patógenos transmitidos por vía sanguínea, como los virus de la hepatitis B y C.^7,8^ De igual forma, este hecho plantea un riesgo para la propia salud de las PVVS, dado que el odontólogo no podrá proporcionar un tratamiento clínico adecuado^13^ y pudiere prescribir algún fármaco que potencie o antagonice con la terapia antirretroviral.^5,10,11^

De acuerdo con el Informe Global 2013 del Programa Conjunto de las Naciones Unidas sobre el VIH/sida, 61.0% de los países informaron de la existencia de leyes contra la discriminación que protegen a las PVVS. En México, con base en lo establecido en el artículo primero constitucional federal y el artículo 1, párrafo segundo, fracción III de la ley federal para prevenir y eliminar la discriminación, se considera un acto contrario a derecho estigmatizar y negar derechos a las PVVS. Por consiguiente, se considera discriminatorio y antiético negar la atención odontológica a las PVVS y a otros usuarios con enfermedades infectocontagiosas puesto que ello vulnera garantías primordiales de los seres humanos como lo es la atención médica eficiente. Sin embargo, con frecuencia, la falta de servicios legales accesibles resulta en que muchos casos de discriminación, relacionados con el VIH, nunca se aborden.

Ambas realidades (el usuario no notifica su serodiagnóstico y el odontólogo niega la atención al usuario infectocontagioso) no garantizan que los odontólogos o sus usuarios eviten la exposición al VIH y a otros patógenos, ya que los individuos que viven con VIH o con alguna otra enfermedad infectocontagiosa pueden no ser conscientes de estar infectados. Por tal motivo, los odontólogos y todos los profesionales de la salud tienen la responsabilidad profesional y ética de capacitarse, sensibilizarse y certificarse en el manejo del VIH y otras enfermedades infectocontagiosas, así como en estudios de género, diversidad y derechos humanos, para mantener altos estándares profesionales y asegurar que todos los usuarios reciban un trato digno y equitativo.
